# A Conceptual Model of Engagement Profiles Throughout the Decades of Older Adulthood

**DOI:** 10.3389/fpsyg.2019.02535

**Published:** 2019-11-14

**Authors:** Kelly Carr, Patti Weir

**Affiliations:** Faculty of Human Kinetics, University of Windsor, Windsor, ON, Canada

**Keywords:** aging, active, positive, multidimensional, activity

## Abstract

Engagement with life is essential to successful aging. This study explored ‘how’ and ‘why’ engagement profiles change throughout older adulthood using a mixed methods design. Fifty-four participants (mean age = 79.17 years, age range = 65–97 years; 21 males, 33 females) completed questionnaires to quantify ‘past’ and ‘present’ engagement. Focus groups and semi-structured interviews were completed with a subsample of participants (*n* = 42). Results highlight participation in a variety of activities across the decades of older adulthood, and identify that engagement in productive and active leisure pursuits decreased in frequency with increasing age, while the frequency of social and passive leisure activities remained stable. Changes in engagement were a function of five themes derived from the fundamental qualitative description: (a) health and physical limitations, (b) death, (c) freedom, (d) desire, and (e) external influential factors. Patterns of engagement frequency are interpreted in consideration of qualitative findings, creating an integrated discussion of ‘how’ and ‘why’ activity profiles emerge during older adulthood. This study highlights the value of a mixed methods approach when examining engagement in older adulthood, and provides practical implications for practitioners who seek to support a successful aging process.

## Introduction

Over the last 50 years there has been a significant investment in research on successful aging (SA), where it has been defined as both an outcome and a process (Baltes and Carstensen, 1996; Fisher and Specht, 1999). While there has been no shortage of research effort devoted to defining the term SA, literature has failed to identify a single, universal definition (Rowe and Kahn, 1987, 1997; Peel et al., 2004; Phelan et al., 2004; Bowling and Dieppe, 2005; Depp and Jeste, 2006, 2009; Cosco et al., 2014; Martin et al., 2015). However, regardless of how SA has been defined, it has benefited the aging process by consistently providing a positive overtone.

Several conceptualizations of SA have been presented in the research literature. As an example of an outcome-oriented approach, Rowe and Kahn’s model defines SA as the intersection of three components: low probability of disease and disease-related disability; high cognitive and physical functioning; and engagement with life. This model stimulated debate regarding the existence of individual control over the ability to age successfully. To illustrate, factors such as diet, exercise, and smoking contribute significantly to disease-related processes. Therefore, by managing a healthier lifestyle, it may improve the odds of SA. Despite its theoretical nature, this model is testable as it is possible to determine whether “success” is present in any, or all three components, thereby defining that individual as successful. Early criticisms of this model commented that its dichotomous approach to defining SA failed to address the heterogeneity of aging individuals, and that it did not account for psychosocial influences on aging. While many studies attempted to identify predictors of successful aging (e.g., life satisfaction, involvement in games and physical activity, income), the establishment of a single comprehensive model, or testable definition of SA has been elusive.

In addition to objective efforts to measure SA, subjective accounts of the aging process have posited that many older adults believe that they are aging successfully despite the presence of medical issues. Strawbridge et al. (2002) reported that 18.8% of older adults were aging successfully when objectively assessed against measures aligned with Rowe and Kahn’s model, while 50.3% subjectively believed they were aging successfully. Similarly, Montross et al. (2006) reported that 92% of respondents rated themselves as aging successfully. These subjective ratings were positively correlated with resilience, activity level, and number of close friends, which suggests a relationship between perceptions of successful aging and engagement with life, and supports the importance of examining both process and outcome as they relate to SA. Evidently, when provided with the opportunity to assess one’s personal level of success, many older adults believe they are successful, imparting a positive overtone on the aging process.

Mathematical modeling of SA has been limited, with the existing studies employing confirmatory factor analysis and structural equation modeling to identify a multidimensional perspective of SA. In their models, Lee et al. (2011) and Geard et al. (2018) included a social factor (i.e., engagement with friends/family) which promoted SA. Similarly, SA was also promoted by a leisure factor (i.e., exercise and vacation time) included within the model proposed by Lee et al. (2011). These studies are the first to mathematically model engagement, as defined by social and leisure factors.

Historically, engagement with life has been the most understudied component of Rowe and Kahn’s original theoretical model, which is evidenced by only 13–26% of definitions of SA including a social and/or engagement measure (Bowling and Dieppe, 2005; Depp and Jeste, 2006). Engagement has been conceptualized in narrow and broad terms, and has been described as an outcome measure, and an antecedent of SA (Menec, 2003; Pruchno et al., 2010). While a host of variables related to engagement have been used as predictors of successful aging (games/sports – Menec, 2003; visiting friends/reading/watching television – Montross et al., 2006), very little is known about the changing nature of engagement, with most studies employing a cross-sectional research design. In addition, it is a unique component of SA, compared to health and functioning, as it is modifiable (Everard et al., 2000) and exists in a variety of forms (i.e., passive leisure, productive activities).

Early definitions of engagement focused on interpersonal relationships and continued participation in productive activities (Rowe and Kahn, 1997). However, definitions have recently been expanded to include regenerative (e.g., eating), passive leisure (e.g., reading), active leisure (e.g., walking), social (e.g., visiting, travel), and instrumental activities (e.g., shopping, cleaning) (Bath and Deeg, 2005; Maier and Klumb, 2005; Mendes de Leon, 2005; Liffiton et al., 2012). In a recent qualitative study, Carr and Weir (2017) identified that three primary themes consistently defined successful aging across three decades of older adulthood (65 to 74, 75 to 84, 85+ year olds): staying healthy, active engagement in life, and keeping a positive outlook on life; again, highlighting the importance of engagement to ongoing success. Despite a broadening of activities that represent engagement, and the recognition by older adults that engagement is an important component of successful aging, previous work has not identified whether the types of activities older adults participate in change across stages of older adulthood. For a comprehensive examination, the present study uses the term ‘engagement’ as an all-encompassing concept for the various elements of active engagement in life (i.e., interactions with others and participation in activities), as it is not limited by terms such as ‘social’ and ‘productive’ (Mendes de Leon, 2005).

Regardless of the nomenclature subscribed to, literature suggests that engagement provides a unique and essential component to various models of successful aging. Thus, from both a research and a practical standpoint, it may be advantageous to understand the demographics of older adults who tend to maintain an active engagement in later life despite age-related changes. Baltes and Carstensen (1996), who provide a process-oriented approach to SA, suggest that relying solely on normative outcomes (e.g., how quickly you can rise from a seated position) limits an understanding of the heterogeneity among and within older adults. Like Salthouse (1991), who referenced accommodation, remediation, and compensation as strategies for dealing with cognitive decline, Baltes and Carstensen (1996) defined successful in relative terms as “the attainment of goals which can differ widely among people and can be measured against diverse standards and norms” (p. 399). They were interested in the processes by which older adults achieved success in a time where there were increasing limitations in psychological, biological, and social resources. They introduced a metamodel of SA incorporating a lifespan view that focuses on the processes of selection, compensation, and optimization to conceptualize the strategies older adults use to age well in the face of increasing losses and limitations (Baltes and Baltes, 1990).

By adopting a lifespan view it is possible to rely on both outcome- and process-oriented views of SA to understand changes in engagement over the decades of older adulthood. The benefits of ongoing engagement include reduced mortality risk (Bassuk et al., 1999; Mendes de Leon et al., 2003; Menec, 2003; Glass et al., 2006), improved physical and cognitive function (Bassuk et al., 1999; Everard et al., 2000; Seeman et al., 2001; Bath and Gardiner, 2005; Bennett, 2005), and psychological health benefits (Seeman et al., 2001; Menec, 2003; Glass et al., 2006). In the present study, employing a mixed methods approach allowed engagement to be examined as both an outcome and process, with the integration of results creating a fuller understanding of the changing nature of engagement across a variety of productive, social, active leisure, and passive leisure activities. The over-arching goal was not only to identify activity profiles and changes in frequency of participation, but the underlying reasons why those changes might occur.

## Materials and Methods

### Participants

A cross-sectional sample of 54 community-dwelling older adults (67–95 years; *M* age = 79.17 years), divided across three decades of older adulthood (*n* = 21, 65–74 years; *n* = 21, 75–84 years; *n* = 12, 85 + years – 21 males, 33 females) were recruited from local organizations, including a senior’s activity center, walking group, exercise class, and church, and ultimately by ‘word of mouth’ in which information regarding the study was provided informally (e.g., by a friend). Given that the participants were community-dwelling older adults, the study sample reflects an aging population that had maintained some level of engagement in a variety of activity types. To further broaden recruitment, snowball sampling was employed allowing participants outside the specific recruitment locations to participate. Additional demographic information about the study sample can be found in Table 1 (see also Carr and Weir, 2017). All participants completed Part 1 assessing ‘how’ engagement had changed. A subsample of participants (*n* = 42; mean age = 79.6 years; *n* = 17, 65–74 years; *n* = 17, 75–84 years; *n* = 8, 85 + years; 19 males, 23 females) from Part 1 were recruited to participate in Part 2 assessing ‘why’ engagement had changed. Interest to complete Part 2 of the research study was indicated by participants on their participant profile, each of whom were then contacted by the primary investigator. Recruitment for Part 2 of the study (qualitative analysis) was stopped once data saturation was met.

**TABLE 1:** Participant demographics included in ‘Part 1: Quantitative Analysis’ and ‘Part 2: Qualitative Analysis.’

**Variable**	**65–74 year olds**		**75–84 year olds**		**85 + year olds**		**Total sample**	
				
**Study**	**1**	**2**	**1**	**2**	**1**	**2**	**1**	**2**
Mean Age (sample size) Range (years)	70.3 (*n* = 21) (65–74 years)	70.1 (*n* = 17) (65–74 years)	78.4 (*n* = 21) (75–84 years)	78.4 (*n* = 17) (75–84 years)	89.2 (*n* = 12) (85–97 years)	90.6 (*n* = 8) (87–97 years)	79.2 (*N* = 54)	79.6 (*N* = 42)
**Sex**								
Male	7 (33.3%)	7 (41.2%)	9 (42.9%)	8 (47.1%)	5 (41.7%)	4 (50.0%)	21 (38.9%)	19 (45.2%)
Female	14 (66.7%)	10 (58.8%)	12 (57.1%)	9 (52.9%)	7 (58.3%)	4 (50.0%)	33 (61.1%)	23 (54.8%)
**Highest level of education**								
Elementary school	1 (4.8%)	0 (0%)	1 (4.8%)	1 (12.5%)	2 (16.7%)	1 (12.5%)	4 (7.4%)	2 (4.8%)
High school	8 (38.1%)	8 (47.1%)	11 (52.4%)	4 (50%)	5 (41.7%)	4 (50%)	24 (44.4%)	21 (50.0%)
College	4 (19.0%)	3 (17.6%)	5 (23.8%)	2 (25.0%)	4 (33.3%)	1 (25.0%)	13 (24.1%)	9 (21.4%)
University	4 (19.0%)	2 (11.8%)	4 (19.0%)	1 (12.5%)	1 (8.3%)	1 (12.5%)	9 (16.7%)	6 (14.3%)
Post-graduate	4 (19.0%)	4 (23.5%)	0 (0.0%)	0 (0%)	0 (0.0%)	0 (0%)	4 (7.4%)	4 (9.55)
**Household Income**								
≤$20,000	0 (0.0%)	0 (0%)	3 (14.3%)	2 (11.8%)	2 (16.7%)	2 (25.0%)	5 (9.3%)	4 (9.5%)
≤$40,000	7 (33.3%)	5 (29.4%)	1 (4.8%)	1 (5.9%)	4 (33.3%)	2 (25.0%)	12 (22.2%)	8 (19.0%)
≤$60,000	3 (14.3%)	3 (17.6%)	3 (14.3%)	1 (5.9%)	1 (8.3%)	1 (12.5%)	7 (13.0%)	5 (11.9%)
≤$80,000	1 (4.8%)	1 (5.9%)	4 (19.0%)	4 (23.5%)	1 (8.3%)	1 (12.5%)	6 (11.1%)	6 (14.3%)
>$80,000	2 (9.5%)	2 (11.8%)	1 (4.8%)	1 (5.9%)	0 (0.0%)	0 (0%)	3 (5.6%)	3 (7.1%)
Prefer not to answer	8 (38.1%)	6 (35.3%)	9 (42.9%)	8 (47.1%)	4 (33.3%)	2 (25.0%)	21 (38.9%)	16 (38.1%)
**Living environment**								
House	15 (71.4%)	14 (82.4%)	15 (71.4%)	17 (100%)	9 (75.0%)	7 (87.5%)	39 (72.2%)	38 (90.5%)
Apartment/condominium	6 (28.6%)	3 (17.6%)	6 (28.6%)	0 (0%)	2 (16.7%)	1 (12.5%)	14 (25.9%)	4 (9.5%)
Retirement residence	0 (0.0%)	0 (0%)	0 (0.0%)	0 (0%)	1 (8.3%)	0 (0%)	1 (1.9%)	0 (0.0%)
**Living arrangement**								
With spouse/partner	10 (47.6%)	7 (41.2%)	14 (66.7%)	13 (76.5%)	2 (16.7%)	2 (25.0%)	26 (48.1%)	22 (52.4%)
With family	2 (9.5%)	2 (11.8%)	2 (9.5%)	1 (5.9%)	2 (16.7%)	0 (0%)	6 (11.1%)	3 (7.1%)
Alone	9 (42.9%)	8 (47.1%)	5 (23.8%)	3 (17.6%)	8 (66.7%)	6 (75.0%)	22 (40.7%)	17 (40.5%)

*Percent values represent percentage of the sample within separate decades of life.*

### Procedure

#### ‘How’

This component was evaluated quantitatively by having participants complete two questionnaires aimed at assessing their current and past (5-years ago) engagement in productive (volunteer work, light housework, care for others, employment, etc.), social (family/friends, visiting others, church related activities, etc.), passive leisure (reading, bingo/games, musical instrument, etc.), and active leisure (walking, moderate sports, strenuous sports, exercise, etc.) activities. The questionnaires consisted of 30 identical items and differed by instructions. The “present engagement” questionnaire asked participants to report current levels of engagement, while the “past engagement” questionnaire asked participants to report levels of engagement 5 years ago. It is important to note that retrospective methods of data collection in older adults are evidenced to be an accurate source of information (Blair et al., 1991; Falkner et al., 1999; Klumb and Baltes, 1999; Young et al., 2008; MacDonald et al., 2009). Both questionnaires utilized a four-point Likert scale to determine weekly participation: 0 times per week, 1–2 times per week, 3–4 times per week, and 5–7 times per week. The ‘Present Engagement Questionnaire’ also required participants to categorize each of the 30 activities as either productive, social, active leisure, or passive leisure. To ensure consistent conceptualization of each term, theoretically-based definitions of each activity type were provided to participants.^1^ Binomial probability analysis was used to determine the activity categorization. Where the analysis was not significant, categorization was achieved through consensus in the focus groups.

#### ‘Why’

Participants aged 65–84 years participated in one of six structured focus groups segregated by decade of older adulthood to allow for homogeneity within a focus group relating to life experiences to date. Three focus groups were conducted with 65 to 74 year olds (*n* = 17) and three focus groups were conducted with 75 to 84 year olds (*n* = 17; detailed demographic information separated by decade of life is provided in Table 1). Five to six participants were included in each focus group, which were audio recorded and approximately 75 to 120 min in duration (Kitzinger, 1995). To meet the needs and/or preferences of the participants, focus groups were conducted in convenient locations (e.g., senior center, church, etc.). Each focus group progressed through an ice breaker question where participants identified a favorite activity, followed by a ‘discussion-starter’ which required participants to record and discuss five reasons they valued engaging in activity (Morgan, 1997). Next, participants completed a group activity. Cards identifying the 30 activities included on the ‘past’ and ‘present’ engagement questionnaires were provided. Within each focus group, participants were required to come to a consensus regarding the categorization of each activity as productive, social, active leisure, or passive leisure. This activity led into the main discussion regarding ‘why’ engagement profiles changed throughout older adulthood. After all the questions in the structured focus group guide had been discussed, the final question addressed any remaining thoughts, concerns, or viewpoints. To increase interviewer reliability and richness of data collected, one pilot focus group was conducted (Appleton, 1995).

In addition to focus groups, semi-structured interviews were completed with a random sample of four participants from each of the two age groups (65 to 74, 75 to 84 year olds), as such a data collection method can provide in-depth insight on themes derived from focus group research (Morgan, 1997). Interviews facilitated participation for individuals 85 years of age and older who were dependent on others for direct care and transportation. Therefore, one-on-one interviews were used as a substitute for focus groups among individuals in the oldest decade of adulthood (*n* = 8). The semi-structured interviews were conducted at a location selected by the participant, were audio recorded, and lasted approximately 15 to 30 min. Semi-structured interviews for the participants 85 years of age and older followed the standardized interview guide developed for the focus groups, except for the group activity. Semi-structured interviews conducted as a follow-up to focus groups used individualized interview guides based on participants’ responses to the ‘past’ and ‘present’ engagement questionnaires. Flexibility within the semi-structured interviews was permitted using appropriate probes to extract the greatest amount of data from each participant (Fossey et al., 2002).

### Data Analysis

#### ‘How’

To determine differences in participation between activity types, the 30 activities identified on the ‘past’ and ‘present’ engagement questionnaires were grouped according to the activity categorization determined by the participants (Table 2). Participant responses on the ‘past’ and ‘present’ engagement questionnaires were coded so that higher numbers represented a greater frequency of weekly participation (1 = 0 times per week, 2 = 1–2 times per week, 3 = 3–4 times per week, 4 = 5–7 times per week); therefore, if frequency of participation was 2.5 this would correspond to 1–4 times per week. Differences were examined through a mixed design 3 (Age) × 2 (Time) × 4 (Activity Type) ANOVA with repeated measures on ‘time’ and ‘activity type.’ To examine differences in participation within each activity type four separate mixed design ANOVAs were conducted to include one analysis for each activity type. These varied based on the number of activities per category: productive (*n* = 9); social (*n* = 8); active leisure (*n* = 7); passive leisure (*n* = 6). Significant *F*-values were determined at a *p*-value of 0.05. Greenhouse–Geisser correction was employed for all analyses as sphericity was violated. Only *F*-values having at least a small effect size (${\text{η}}_{\text{p}}^{2}$ ≥ 0.01) were analyzed to ensure the presence of practical significance within the associated effect (Cohen, 1988). Significant interactions were examined using analysis of simple effects. Where applicable, significant *F*-values were *post hoc* tested through pairwise comparisons and evaluated based on Bonferroni’s adjustment for multiple comparisons.

**TABLE 2:** Categorization of specific activities into four activity types.

**Activity**	**Binomial probability**	**Focus group**	***N***
**Productive activities**			
Volunteer work		X	52
Light housework	X		53
Care for others		X	52
Educational activities	X		53
Playing a musical instrument		X	48
Full- or part-time paid employment	X		49
Home repairs	X		51
Heavy housework		X	51
Service, club, or fraternal organization activities		X	51
**Social activities**			
Family/friendship activities	X		53
Visiting others	X		53
Cultural activities		X	52
Church-related activities		X	50
Bingo, cards, or other games	X		51
Attending theater events		X	52
Neighborhood or community activities		X	52
Phone conversations	X		53
**Active leisure activities**			
Moderate sports or recreational activities		X	52
Outdoor gardening, sweeping the balcony or stairs		X	53
Strenuous sports or recreational activities	X		52
Exercise to increase muscle strength and endurance		X	52
Taking a walk outside your home or yard		X	53
Light sports or recreational activities		X	52
Lawn work or yard care		X	51
**Passive leisure activities**			
Watching television	X		53
Handicrafts		X	52
Reading	X		53
Listening to the radio or music	X		53
Computer activities		X	50
Crosswords, puzzles, etc.	X		51

*N = total sample size of participants that categorized each specific activity.*

#### ‘Why’

Data from both the focus groups and semi-structured interviews were transcribed verbatim and corrected against audiotapes. Focus group and interview transcripts remained separate during the analysis to allow unique themes to emerge due to the different social contexts under which data were collected (i.e., group setting versus individual setting). This was an important consideration as the differing data collection methods had the potential to influence participants’ responses, such as through the promotion of socially acceptable answers during focus groups or sharing of intimate details during one-on-one interviews. Thus, group influences were considered when analyzing focus group responses and therefore, maintaining separation between data collection approaches allowed comparisons of themes that did, or did not emerge, due to contextual factors (Smithson, 2000).

Data analysis included qualitative content analysis (Sandelowski, 2000) to provide a coherent organization of consistencies within qualitative data (Patton, 2002). Based on the transcribed data, relevant information was highlighted to create broad themes among responses. Using an inductive approach, *meaning units* were developed from the specific responses within these broad themes (Tesch, 1990; Côté et al., 1993). This process occurred within and between focus groups and semi-structured interviews (Maykut and Morehouse, 1994). Subsequently, through continuous comparisons and organizations, distinct themes based on commonalities of *meaning units* were created (Tesch, 1990; Côté et al., 1993). This constant comparative method continued until no new themes were identified and data saturation was achieved (Taylor and Bogdan, 1984; Ryan and Bernard, 2003). Successful attainment of data saturation and replication, along with concurrently engaging in data collection and analysis, supported the rigor of the qualitative data analysis (Morse et al., 2002). This analytical approach also ensured that the themes that emerged remained ‘close’ to the data (Taylor and Bogdan, 1984; Sandelowski, 2000). Comparisons of the themes across qualitative methods (i.e., focus groups versus interviews) resulted in collapsing all data together, as no new themes emerged from semi-structured interviews. Similarities and differences between decades of life (i.e., 65–74, 75–84, 85 + years of age) were also examined and identified where applicable. In total, 13.5 h of audio recording produced 278 pages of transcripts to be analyzed. From these transcripts, 240 meaning units were identified, which yielded 5 themes and 17 subthemes. Methodological rigor was further established through the review of the data analysis by the senior author, who independently categorized meaning units into higher order themes. This led to a slight reorganization of meaning units and themes to achieve agreement between the two investigators. Where agreement could not be attained, the meaning unit was removed as it did not provide reliable data (Morse et al., 2002; Patton, 2002). Additionally, researcher biases were systematically documented through note-taking during data analysis, as such explicit awareness may reduce biases from influencing results (Morse and Richards, 2002).

## Results

### ‘How’

#### Part 1: Quantitative Analysis

**FIGURE 1:**
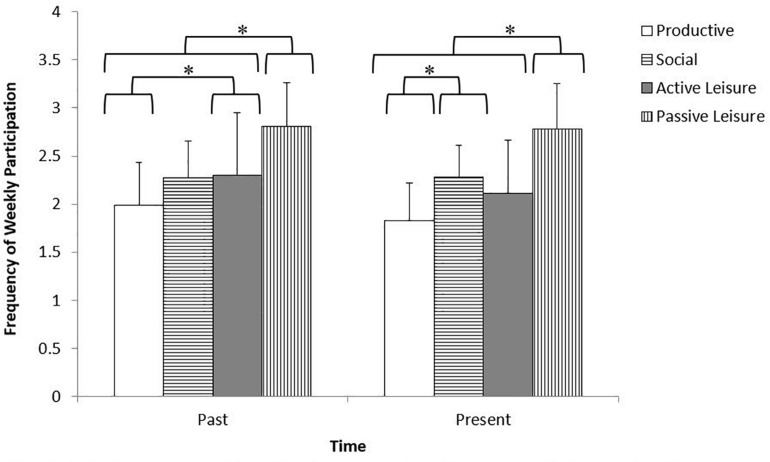
Frequency of weekly participation in activity types separated by time. This figure illustrates the frequency of weekly participation in each activity type separated by time for adults 65 years of age and older. Scale for frequency of weekly participation corresponds to Likert scale provided on questionnaires (1 = never; 2 = seldom; 3 = sometimes; 4 = often); error bars represent standard deviation. ^∗^*p* ≤ 0.05.

The examination of frequency of weekly participation across the four activity types revealed a significant interaction between time and activity type [*F*(2.63,134.19) = 4.48, *p* = 0.007, ${\text{η}}_{\text{p}}^{2}$ = 0.081]. In order to determine whether the pattern of participation across activity types was consistent, separate one-way ANOVAs were conducted comparing activity type in the past, [*F*(3,215) = 25.87, *p* = 0.000, ${\text{η}}_{\text{p}}^{2}$ = 0.268], and in the present, [*F*(3,215) = 42.90, *p* = 0.000, ${\text{η}}_{\text{p}}^{2}$ = 0.378]. In the past (i.e., 5 years ago) weekly participation in passive leisure activities (*M* = 2.81, *SD* = 0.45) was significantly greater than the frequency of participation in any other activity type (productive: *M* = 1.99, *SD* = 0.44; social: *M* = 2.27, *SD* = 0.39; active leisure: *M* = 2.30, *SD* = 0.65); and, the frequency of weekly participation in active leisure activities was greater than that of productive activities. Similarly, in the present, passive leisure activities (*M* = 2.78, *SD* = 0.47) were participated in more often than all other activity types (productive: *M* = 1.83, *SD* = 0.39; social: *M* = 2.28, *SD* = 0.33; active leisure: *M* = 2.11, *SD* = 0.56), while participation in social activities was greater than participation in productive activities. Figure 1 illustrates the frequency of past and present weekly participation for each activity type.

This analysis also revealed a main effect of age, [*F*(2,51) = 4.86, *p* = 0.012, ${\text{η}}_{\text{p}}^{2}$ = 0.160], where 65–74 year olds (*M* = 2.42, *SD* = 0.45) reported higher weekly participation when compared to participants who were 85 years of age and older (*M* = 2.09, *SD* = 0.45). However, on average, both age cohorts participated in activity between one and four times a week. In addition, there was a main effect of time [*F*(1,51) = 7.31, *p* = 0.009, ${\text{η}}_{\text{p}}^{2}$ = 0.125], which identified more frequent weekly participation in the past (*M* = 2.31, *SD* = 0.34) compared to the present (*M* = 2.22, *SD* = 0.30). While a main effect of activity type was also evidenced [*F*(2.48,126.62) = 60.13, *p* = 0.000, ${\text{η}}_{\text{p}}^{2}$ = 0.541], this factor was included in the interpretation of the significant interaction and was not further analyzed.

##### Productive activities

Weekly participation in productive activities revealed a significant interaction between time and activity [*F*(6.12,312.00) = 3.78, *p* = 0.001, ${\text{η}}_{\text{p}}^{2}$ = 0.069]. Results from a series of repeated measures ANOVAs examining the difference between past and present weekly participation for each productive activity are reported in Table 3. Specifically, the frequency of weekly participation for five productive activities (volunteer work, care for others, employment, home repairs, heavy housework) was significantly lower in the present compared to the past, while the remaining four productive activities experienced no change in the frequency of weekly participation over time. This analysis also revealed a significant main effect of age [*F*(2,51) = 6.54, *p* = 0.003, ${\text{η}}_{\text{p}}^{2}$ = 0.204]. It was determined that 65–74 year olds (*M* = 2.10, *SD* = 0.96) reported a significantly higher frequency of weekly participation in productive activities when compared to participants 85 years of age and older (*M* = 1.55, *SD* = 0.75). Additionally, there was a main effect of time, [*F*(1,51) = 10.00, *p* = 0.003, ${\text{η}}_{\text{p}}^{2}$ = 0.164], which identified a decrease in the frequency of weekly participation in productive activities from the past (*M* = 1.94, *SD* = 0.41) to the present (*M* = 1.80, *SD* = 0.38). There was also a main effect of activity [*F*(6.70,341.54) = 28.85, *p* = 0.000, ${\text{η}}_{\text{p}}^{2}$ = 0.361], however, this factor was included in the interpretation of the significant two-way interaction, and therefore was not considered for further analysis.

**TABLE 3:** Past vs. present weekly participation in productive activities among adults 65 years of age and older.

**Specific activity**	**Past Mean (SD)**	**Present Mean (SD)**	***F*-statistic df (1, 53)**	***p*-value**	**${\text{η}}_{\text{p}}^{2}$**
Volunteer work^∗^	2.20 (0.96)	1.94 (0.96)	4.77	0.033	0.082
Light housework	3.24 (0.87)	3.33 (0.91)	0.38	0.540	0.007
Care for others^∗^	2.04 (1.22)	1.72 (1.00)	4.34	0.042	0.076
Educational activities	1.65 (0.91)	1.78 (1.00)	1.60	0.212	0.029
Playing a musical instrument	1.35 (0.87)	1.39 (0.92)	0.16	0.687	0.003
Full- or part-time paid employment^∗^	1.63 (1.14)	1.11 (0.50)	12.94	0.001	0.196
Home repairs^∗^	1.87 (0.91)	1.63 (0.81)	10.45	0.002	0.165
Heavy housework^∗^	2.31 (0.87)	1.89 (0.93)	11.00	0.002	0.172
Service, club, or fraternal organization activities	1.59 (0.86)	1.70 (0.92)	1.13	0.293	0.021

*Weekly participation is based on a four-point Likert scale (1 = never [0 times], 2 = seldom [1–2 times], 3 = sometimes [3–4 times], 4 = often [5–7 times]). ^∗^*p* ≤ 0.05.*

##### Social activities

Participation in social activities indicated a significant main effect of activity [*F*(5.55,282.84) = 54.17, *p* = 0.000, ${\text{η}}_{\text{p}}^{2}$ = 0.515]. Family and friendship activities (*M* = 3.30, *SD* = 0.78) and phone conversations (*M* = 3.24, *SD* = 0.78) were participated in significantly more often on a weekly basis than all other social activities (visiting others, cultural activities, church related activities, bingo/games, attending theater, neighborhood activities). Additionally, participants reported visiting others (*M* = 2.55, *SD* = 0.73) significantly more often than participating in cultural activities, bingo, cards, or other games, theater events, and neighborhood or community activities. No other significant differences were identified between social activities with respect to participants’ frequency of weekly participation.

##### Active leisure activities

Frequency of weekly participation revealed a significant main effect of time [*F*(1,51) = 8.72, *p* = 0.005, ${\text{η}}_{\text{p}}^{2}$ = 0.146]. Participants engaged significantly less often in active leisure activities in the present (*M* = 2.11, *SD* = 1.03) compared to the past (*M* = 2.31, *SD* = 1.04). However, when anchored to the Likert scale, participants engaged in active leisure activities between one and four times per week in both the past and the present. A significant main effect of activity was also identified [*F*(4.52,230.56) = 12.26, *p* = 0.000, ${\text{η}}_{\text{p}}^{2}$ = 0.194]. Taking a walk outside one’s home or yard (*M* = 2.81, *SD* = 0.88) was participated in significantly more often on a weekly basis than all other active leisure activities (moderate sports, strenuous sports, exercise to increase strength, light sports, lawn work), except for outdoor gardening and sweeping the balcony or stairs (*M* = 2.59, *SD* = 1.04). Additionally, weekly engagement in outdoor gardening and sweeping the balcony or stairs was significantly greater than participation in light sports and recreational activities (*M* = 1.73, *SD* = 0.94), and lawn work or yard care (*M* = 1.96, *SD* = 1.07). Average participation in taking a walk outside one’s home or yard, gardening and sweeping the balcony or stairs, and exercise to increase muscle strength and endurance were reported to occur between one and four times per week, whereas all other active leisure activities were reported to be participated in less than twice per week.

##### Passive leisure activities

Participation in passive leisure activities revealed a significant interaction between activity and age [*F*(8.90,226.84) = 2.33, *p* = 0.016, ${\text{η}}_{\text{p}}^{2}$ = 0.084]. Listening to music or the radio was the only passive leisure activity that differed significantly among age groups. Specifically, participants 85 years of age and older (*M* = 2.42, *SD* = 1.24) listened to music or the radio significantly less often than either the 65 to 74-year-olds (*M* = 3.45, *SD* = 0.80), or the 75 to 84-year-olds (*M* = 3.43, *SD* = 0.88). On average, 65 to 84-year-olds listened to music or the radio between 3 and 7 days per week, in contrast to the 1 to 4 days per week for participants 85 years of age and older. While listening to the music or radio decreased as a function of age, the remaining passive leisure activities experienced no change. A main effect of activity [*F*(4.45,226.84) = 30.83, *p* = 0.000, ${\text{η}}_{\text{p}}^{2}$ = 0.377] was not analyzed further as it was included in the interpretation of the two-way interaction.

### ‘Why’

#### Part 2: Qualitative Analysis

A sub sample (*n* = 42) of the participants included in ‘Part 1: Quantitative Analysis’ were involved in the qualitative procedures and analysis. Analysis of focus group and semi-structured interview data yielded five themes related to ‘why’ engagement profiles change throughout older adulthood: (a) health and physical limitations, (b) death, (c) freedom, (d) desire, and (e) influential external factors. As displayed in Table 4, several subthemes existed within each theme.

**TABLE 4:** Themes and subthemes identified as reasons for changes in engagement profiles over the previous 5 years of older adulthood.

**Theme/subtheme**	**Meaning unit**	**65–74**	**75–84**	**85 +**
**Health And Physical Limitations**				
Unspecified health issues and physical limitations	“It all boils down to health issues because we can’t do as much as we used to” (FR, 75–84 years).	X	X	X
Specified health issues and physical limitations	“I have arthritis and I can’t run, can’t ride my bike, I can’t even garden well because I can’t bend over like I used to do… I would say arthritis has made a big difference to me as far as my active leisure” (CR, 75–84 years).	X	X	X
Decreased physical senses	“With my lack of eyesight I can’t do the things that I used to do” (OL, 75–84 years).	X	X	X
Decreased energy levels	“Five years ago I could work for 2 or 3 h at a time, now I can work for 20 min to a half hour. Because I’m getting older… your energy seems to evaporate” (OV, 85 + years).	X	X	X
Progressive ‘slowing’	“As you go along in life you just naturally slow down” (HU, 85 + years).	X	X	X
Combating health issues and physical limitations	“I’m more active now than I was 5 years ago, it’s a way of combating arthritis” (IY, 75–84 years).	X	X	
**Death**				
Death in social circle	“My social network has shrunk considerably… within the last 5 years, shrunk because of death” (RR, 75–84 years).	X	X	X
Death of spouse	“I don’t have a husband anymore… that changes your life completely. There are a lot of things that you do as a couple that you won’t do by yourself” (UF, 85 + years).	X	X	X
**Freedom**				
Freedom of time	“You’re doing things you never had time for, maybe you thought about doing but you couldn’t” (FD, 65–74 years).	X	X	
Freedom of choice	“You pick and choose your activities a bit more than perhaps you did before because you’re not socially obligated to do things that you may have been before” (LL, 65–74 years).	X	X	
Freedom from past priorities	“Now that the work and other things that were priorities are not, I don’t have to worry about them, so I can focus more on what I need” (ED, 65–74 years).	X		
Freedom from ‘sweating the small stuff’	“I think as I’m getting older, I don’t ‘sweat the small stuff.’ You don’t get upset about little things as much and you focus on what’s really important. You don’t care what people think as much, you’re a little bit more laid back, you don’t worry about things” (LL, 65–74 years).	X	X	
**Desire**				
Lack of desire	“You just don’t have the desire… to participate in a physical manner… it’s just I sit in the chair and I don’t want to get up” (YO, 75–84 years).	X	X	X
Change in what is desired	“Our ideas change, what we used to like as leisure we don’t care for anymore, we want to do something different” (LU, 75–84 years).		X	X
**Influential External Factors**				
Family role	“Changes in the family… I have a granddaughter and a grandson… that really changes your whole priority of what you do, your life is entirely different when the grandkids come along” (LL, 65–74 years).	X	X	
Finances	“Some people…simply can’t afford on a limited income to participate like some of us do. You’re going all the time and you have enough funds to enjoy that but a lot of people around, it’s strictly money” (DO, 75–84 years).	X	X	
Availability of direct support	“There’s a neighbor that’s kind enough to cut my grass and shovel my snow, so I don’t have to worry about that” (CO, 85 + years).		X	X

##### Health and physical limitations

Nearly all participants discussed personal health status and limitations to physical functioning as affecting engagement in activity over the previous 5 years. Within this theme, six subthemes emerged: (a) unspecified health issues and physical limitations, (b) specified health issues and physical limitations, (c) decreased physical senses, (d) decreased energy levels, (e) progressive ‘slowing,’ and (f) combating health issues and physical limitations.

###### Unspecified health issues and physical limitations

Participants within all decades of older adulthood identified a decrease in activity because of unspecified health issues and physical limitations. Some participants, such as OU, simply blamed ‘health’ for a decrease in specific activities: “your health stops you from doing a lot of things, like driving, I drove and I can’t anymore” (75–84 years). Other participants, such as OH, used the broad explanation of one’s ‘body’ as the cause of a decrease in all activity: “I can’t seem to do things I used to, my body won’t let me” (85 + years).

###### Specified health issues and physical limitations

Participants discussed a general sense of limitation that resulted from a specific health issue: “I have an existing heart problem, so it doesn’t allow me to do what I want to do, so there’s a lot of restrictions as you age” (EG, 65–74 years). Distinct age-related differences were expressed as younger participants often spoke of specific health conditions affecting participation in sport and recreation, such as YU: “I had to get both hips replaced and that took care of the hockey and the basketball” (65–74 years). In contrast, participants within the oldest decade of adulthood focused more on fundamental activities, such as the ability to walk: “I don’t [walk] much, I can when I have to, like we go grocery shopping and I’ll trudge along the store leaning on a cart… I have peripheral neuropathy, nerve damage to the legs, doesn’t let me walk anymore” (HU, 85 + years).

In addition to specified health issues, participants in all decades of older adulthood identified three specific physical limitations: (a) decreased strength, (b) decreased balance, and (c) pain. Decreased strength was acknowledged by participants such as GY: “we’re not as strong physically… that’s the truth of the matter, you think you’re strong, but we’re not as strong physically as we were” (75–84 years). Similarly, a lack of balance was recognized by participants: “our balance isn’t what it was 5 years ago” (FR, 75–84 years), and was further discussed as limiting participation in activity: “they switched some of my blood pressure medication which destroyed my sense of balance… that means that I’m not able to do the walking I used to do” (OV, 85 + years). Finally, pain was associated with limiting participation in personally meaningful activities by participants like EG: “My dancing has changed because it’s a lot more painful than I’ve ever imagined” (65–74 years). This extended to participants in the oldest decade of life, such as FC, who stated “I don’t go [to church] and sit on those hard chairs because it hurts my back” (85 + years). Overall, participants in all decades of older adulthood acknowledged specific health issues and physical limitations as reasons for decreasing engagement in specific activities.

###### Decreased physical senses

Participants in all decades of older adulthood identified decreases to one’s physical senses as affecting engagement in a variety of activities over the previous 5 years. Reductions to the sense of vision was experienced by participants of all ages and created limitations for engagement in various activities. For example, EG, no longer participated in his once-enjoyed handicrafts:

I do upholstery, which I can’t do very readily anymore because my eyesight is not there, I can’t thread a needle anymore, so I find that most of my activities have gone downhill… at one time I loved them… [I can’t] sit at it because I can’t see properly (65–74 years).

Additional passive leisure activities, such as reading, were discussed as being affected by the deterioration of one’s vision: “I have problems with my eyes so reading is not high on my list” (HU, 85 + years). Eyesight was also identified as a factor in losing the ability to drive, which has the potential to broadly affect one’s participation in activity as stated by CO: “I don’t go out much because I can’t drive anymore because of my eyes” (85 + years).

The impact of losing one’s auditory ability was discussed only among participants 75 to 84 years of age. Specifically, participants identified one’s ability to hear as essential to social interactions, as expressed by GR: “[my husband] can hear very little even with two hearing aids, so that has really changed our social interactions” (75–84 years). In addition, the loss of hearing was stated to affect one’s enjoyment during passive leisure activities as explained by TU, “some television programs that I really enjoy and all the other shows, almost anything else on television for me anymore has become impossible, my hearing doesn’t allow me to enjoy it” (75–84 years). Evidently, a decrease in one’s physical senses has far reaching limitations, as participants clearly demonstrated the negative impacts of such losses.

###### Decreased energy levels

Participants in all decades of older adulthood identified decreased participation in active leisure activities, such as EG: “my [active leisure] has gone downhill, the energy levels are not there… things that I did in my past are all over” (65–74 years). This decrease in energy was also associated with a decrease in productive activities as stated by LY, who maintained full-time employment at the time of data collection:

I’m still working full-time…when I come home I don’t do anything, like I don’t vacuum, I don’t paint, I don’t do any of that stuff because I don’t have the energy to do all the stuff that I did 5 years ago after work (65–74 years).

As a consequence, participants indicated decreased energy levels resulting in an increase in the time spent engaging in passive leisure activities, as explained by CR: “you sit and watch TV because you’re tired, you need to rest, it’s not because you’re dying to watch television, and you’re not going to sit there and do nothing, so you turn the TV on” (75–84 years). Interpretation of the data collected alludes to a shift in time-use due to reduced energy levels, where time spent engaging in productive and active leisure activities is replaced by time spent in passive leisure pursuits.

###### Progressive ‘slowing’

Participants often spoke of ‘slowing down’ in a general sense, such as DO: “I used to do things faster, now I do them much slower” (75–84 years). Similarly, DN, applied the notion of ‘slowing down’ to participation in social activities: “[social activities] have gone from young activities to old activities, much slower pace” (75–84 years). It is important to note that many participants, such as LU who quoted her 87-year old husband, demonstrated the maintenance of past activities despite the decrease in the pace in which the activity was completed: “I can do anything that I used to do but it takes me maybe three or four times as long to do it” (75–84 years). Overall, progressive ‘slowing’ with age did not necessarily affect the types of activities participated in, rather completing the activities at a slower pace allowed participants to maintain engagement in specific activities.

###### Combating health issues and physical limitations

This theme was dominated by participants within the youngest decade of older adulthood (i.e., 65–74 years of age). This was articulated by HD:

I think sometimes your health dictates how you change, like I didn’t particularly work out a lot but I ended up having to have heart surgery and they tell you to start working out and keep yourself in shape so suddenly things that you didn’t place as high on the priority list you suddenly say, ‘this is a priority, I have to do this all the time’ (65–74 years).

Similarly, ED, supported this theme with a simple, all-encompassing statement: “active leisure activities do increase because you need it for fitness” (65–74 years). Evidently, individuals in the younger years of older adulthood acknowledged the importance of participating in active leisure activities to combat health issues and physical limitations and changed personal engagement patterns accordingly.

##### Death

All participants acknowledged that engagement over the previous 5 years had been affected by the death of individuals with whom a relationship was shared. This theme included two subthemes: (a) death in social circle, and (b) death of spouse.

###### Death in social circle

Deaths experienced within the participants’ social circles were discussed as limiting social activities throughout all decades of older adulthood. However, this theme became more prominent among participants of older ages. Participant, OF, indicated that she had experienced the death of many friends whom she had once engaged in social activities with: “we had a group of 12 or 14 friends and we only have one couple left, and that’s the thing that is limiting” (65–74 years). This experience was similar among participants in the oldest decade of adulthood, though such participants often expressed the loss of their social circle in its entirety. For example, UF, explained that she no longer had social contacts due to the death of her friends: “I had an awful lot of lovely friends and a very good life, and they’re all dead. When you get really old, everybody that you knew or you liked…all the couples we chummed around with are all gone” (85 + years). This view was further supported through conversation regarding social activities with CI who stated: “I’ve outlived all my friends,” (85 + years) and FC who explained: “I haven’t got any [friends], they’re all dropped off… died” (85 + years).

The theme of ‘death in social circle’ also encompassed participants expressing their concern of their own impending death or the impending death of persons within their social network. However, this concern was only voiced by participants within the younger two decades of older adulthood. Impending death affected one’s participation in social activities as participants often choose to engage in specific social outings to ensure visitation with friends and family prior to the death of either party. This concept was explained by HD:

I think you become more conscientious about your family that you probably didn’t see much when you were working, but now you’re getting older you’re thinking ‘boy, they’re all getting older too, I want to make contact with these people.’ I wouldn’t have thought about that 10 years ago, you just assume they’re going to be there, too many funerals (65–74 years).

Overall, participants in the younger decades of older adulthood viewed impending death as a reason to engage in specific social activities, while participants within all decades of older adulthood (though more pronounced in the older decades) experienced a decrease in social activities following the death of individuals within their social circle.

###### Death of spouse

Following the death of one’s spouse, participant OL expressed an overall reduction in engagement: “5 years ago we volunteered, we worked at the church, we went out every day pretty well… I’m no longer doing that… my life has changed completely” (75–84 years). Other participants spoke of a shift in time-use toward more passive leisure activities following the loss of one’s spouse: “I didn’t read in the day time as much when I was married, we were always on the go, there were always nice things to do” (UF, 85 + years). Participation in activity was also affected after the loss of one’s spouse due to the status of being widowed, or no longer coupled. For instance, OL spoke of reductions in her social activities: “you don’t get invited out as a single person as you do as a couple, I don’t entertain as often because it’s a big effort because you don’t have any support” (75–84 years). Also creating a limitation to participating in social activities was the loss of friendships following the death of one’s spouse, as explained by ET:

“As soon as [your spouse] dies, [your friends] don’t know you anymore. They don’t even phone you, nothing. I couldn’t believe it, and the wives don’t want you to talk to their husbands…I was just all on my own” (75–84 years).

Consequently, the loss of one’s spouse was reported to negatively impact one’s participation in social activities, and resulted in a shifting of time-use toward passive leisure pursuits across all participant groups.

##### Freedom

A change in one’s engagement profile due to a sense of freedom was unique among participants within the younger two decades of older adulthood (i.e., 65–74 and 75–84 years of age) suggesting that recognition of freedom may be a function of the length of one’s time spent in one’s senior years. Younger participants may feel free from the responsibilities and stresses of middle adulthood, while the participants within the oldest decade of adulthood may have become accustomed to this freedom. The theme of freedom encompassed four subthemes: (a) freedom of time, (b) freedom of choice, (c) freedom from past priorities, and (d) freedom from ‘sweating the small stuff.’

###### Freedom of time

Participants under the age of 85 years identified an increase in participation due to an increase in free time. CR discussed an increase in participating in passive leisure activities as the increased availability of free time allowed participants to shift their time to activities they enjoyed: “I read more because I have more time and I absolutely love to read. I love to have a book… before you were so busy it’d take months to read a book” (75–84 years). Likewise, passive leisure activities that were previously considered a ‘waste of time’ had now become enjoyed during the free time of TU: “I have more time to do nothing. In my opinion, reading the newspaper was a waste of time, now I love it. It’s still a waste of time, but I love it” (75–84 years). The freedom of one’s time also allowed participants to increase participation in preferred productive activities in the form of volunteerism: “I started to visit people, shut-ins, so that’s something I didn’t do 5 years ago because I didn’t have the time to do it. I’ve always had the interest but not the time” (IY, 75–84 years). This trend was also extended to family activities: “definitely more time with family, with my grandchild. We’re able to do more because we have more time to do it” (DD, 65–74 years), as well as active leisure activities: “while you’re working you’re sort of mulling along, now that I’m retired I make the time to do exercises and do other things that are more enjoyable” (LL, 65–74 years), and finally, travel: “the big thing is travel… because you can get away… you have the time to travel. I couldn’t take 2 months off to go away on a holiday when I was working 5 years ago*”* (LL, 65–74 years). Evidently, no universal pattern of time-shifting existed among the participants due to the freedom of one’s time, rather participants expressed an individualized pattern of time-shifting toward personally preferred activities.

###### Freedom of choice

Freedom of choice was primarily discussed by participants within the youngest decade of older adulthood (i.e., 65–74 years of age) as a factor that influenced participation in activity over the previous 5 years, but was also mentioned by those between 75 and 84 years. Participants often spoke of the ability to ‘pick and choose’ what activities they wished to participate in, as explained by CB:

I have choices I can make. Prior to retirement I didn’t have as many choices, it was work, which was the priority. Coming home, I was tired at the end of the day so I didn’t get out. Now I can wake up and think, ‘what do I want to do today?’ (65–74 years).

Participants also related the freedom of choice to their social circle; for example, GR explained a change in her social circle due to her freedom to choose her friends: “you’re able to choose who you want to be friends with. When you work with people you’re stuck” (75–84 years). Similar to the freedom of time associated with older adulthood, the freedom of choice was also discussed as an avenue that allowed older adults to experience greater enjoyment throughout their senior years, as explained by NR: “probably [the] quality of [your social circle] improves because you select and do what you want, you meet the people you want to, spend time with fewer people but whatever time you spend you enjoy more” (65–74 years). Therefore, the freedom of choice enabled participants to engage in activities that were personally enjoyed with social contacts they preferred, and thus no common pattern of changing engagement profiles was identified among participants based on one’s freedom of choice.

###### Freedom from past priorities

Freedom from past priorities was frequently and extensively discussed among participants in the youngest decade of older adulthood (i.e., 65–74 years of age) and remained unique to this age group. As expected, participants, such as ED, related freedom from past priorities to retirement: “now that the work and other things that were priorities are not, I don’t have to worry about them” (65–74 years). Past priorities also extended beyond formal employment to additional productive activities, such as housework, which did not hold the same importance as it did in the past as explained by FD:

I am not as concerned with the housework as my leisure. I don’t have as much emphasis on the housework, I don’t really lose any sleep if something is not done, but I do care if I miss my theater or my community choir. So, there’s more enjoyment now in my leisure versus the house that always needs something and at one time it was really important (65–74 years).

This shift in one’s priorities to more personally enjoyed activities was also supported by NV: “now that we’re in our senior years, all that stuff that was so important before like working and house cleaning… now I think more of maybe how much time I have left and I’m going to enjoy it” (65–74 years). Taken together, participants within the youngest decade of older adulthood identified a change in engagement patterns over the previous 5 years through the removal of past priorities which allowed participants the opportunity to prioritize activities based on the enjoyment one received from participation.

###### Freedom from ‘sweating the small stuff’

Participants within the two youngest decades of older adulthood (i.e., 65–74 and 75–84 years of age) indicated a ‘mellowing’ over the past 5 years which was reported to have affected engagement in activity. This concept was articulated by DO: “as I age I find myself becoming less intense about everything… we let things go that we wouldn’t have a few years ago” (75–84 years). This notion of ‘letting things go’ was applied to participation in activities by GO: “I think as you get older you get calmer, you know, ‘don’t sweat the small stuff.’ Things don’t bother you, you’re not trying to cram everything into 24 h that would take 48 h” (65–74 years). This thought was supported by TF:

At one time you were [ready] to do anything, you know ‘I got to do this, let’s do it and get it over with.’ By the time you got that finished there was something else, but today you say, ‘oh well… I’ll wait until the next day’ (65–74 years).

Freedom from ‘sweating the small stuff’ was also reflected in participants’ carefree attitude that had emerged in the previous 5 years. For example, ED explained that she felt freer to engage in different activities:

I’m trying different things because of where I’m at in my life. It’s just like you lose the fear of what you do… maybe it’s good that you do, and you just let go of being so serious all the time (65–74 years).

Overall, a clear change in engagement profiles due to the freedom from ‘sweating the small stuff’ was not revealed, however participants indicated a reduction in pressure to complete specific activities, and a greater willingness to attempt new activities.

##### Desire

One’s desire for participation in activity was acknowledged as a factor that influenced engagement patterns over the previous 5 years for participants of all ages. However, this was more pronounced among the older two decades of adulthood (i.e., 75–84, and 85 + years). The theme of ‘desire’ included two subthemes: (a) lack of desire, and (b) change in what is desired.

###### Lack of desire

Participants of all ages identified a lack of desire as negatively affecting participation over the previous 5 years. Participants, such as CR, often expressed that the lack of desire was rooted in laziness: “some of mine is laziness, I’ll be honest, you just don’t bother” (75–84 years). However, other participants discussed a lack of desire to participate in activity due to changing interests: “I think the interest in things has something to do with it, you may still be able to do it, but you don’t want to do it anymore” (YO, 75–84 years). Some participants spoke of decreased participation in specific activities, such as social outings: “Even going out socially, I don’t have the desire to go out on Fridays dancing because I’m not up to it” (EG, 65–74 years), productive activities: “house repairs, things that have to be done, I don’t want to do them” (FY, 75–84 years), and active leisure pursuits: “it’s too much bother to get up and go for a walk” (TF, 65–74 years). Consequently, participants experienced a decrease in various activities due to a general weakened desire to participate.

###### Change in what is desired

Participants in the two oldest decades of adulthood (i.e., 75–84 and 85 + years of age) indicated that the desire to participate in specific activities had changed over the previous 5 years. Some discussed changes in preferences in a broad sense, such a FY: “you might have developed more interest in some of your leisure activities therefore you might want to do them more than you did before” (75–84 years). This was the case for many participants who expressed a shift in desire to participate more often in passive leisure activities: “I’m more happy just to stay home during the week… it’s like after the whole day you’re ready to just relax at night” (75–84 years). Evidently, this shift in desire translated into greater contentment with participating in home-based activities as expressed by CR who quoted her 82-year old friend: “one thing a week is all I care to do, I’m quite happy to stay home every night” (75–84 years). Evidently, participants within the two oldest decades of adulthood acknowledged that the desire to participate in activities had shifted toward home-based passive leisure activities, and thus affected one’s overall pattern of engagement.

##### External influential factors

Engagement profiles of participants within all decades of older adulthood were affected by external influential factors. Within this theme three subthemes emerged: (a) family role, (b) finances, and (c) availability of direct support.

###### Family role

Participation in activity was thought to be affected by one’s family role for participants in the younger two decades of older adulthood (i.e., 65–74 and 75–84 years of age). Specifically, participants identified that having a role as a grandparent had changed their engagement profiles over the previous 5 years. Some participants, such as CR, expressed an increase in family activities due to the time spent with grandchildren:

We’re involved with our kids a lot, we have nine grandchildren, and I choose sometimes to be around for those kids, I prefer that, I’m just loving the fact that we can be there for them. Sometimes I might not commit to something, I rather be there if the kids come… that’s one of the reasons things have changed (75–84 years).

However, other participants identified a decrease in family activities because their grandchildren were now adolescences and did not require direct care. This concept was explained by FD: “some of my family time has decreased because I don’t have to babysit as much, my granddaughters are older now, so I’m not called upon to put on the bus, take off the bus, that kind of thing” (65–74 years). Therefore, the role that participants held within their families had the potential to change engagement profiles depending on the participants’ responsibilities as a grandparent.

###### Finances

Participants within the younger two decades of older adulthood (i.e., 65–74 and 75–84 years of age) identified finances as a factor that affected participation in activity. Some participants, such as DN, spoke of specific instances where one’s financial situation dictated participation in activity:

When we go out with my Red Hat Group we usually go to either lunch or dinner… we have to be careful because everybody’s on a fixed income and some of them really have to be careful, so we plan where we’re going to go to eat that has to be reasonable (75–84 years).

The importance of finances to one’s participation in social activities was further supported by OF in her response to why her social activities had changed: “money, if you’re a widow you have to take care of money yourself” (65–74 years). As one would expect, financial security aided in the ability to participate in activity throughout older adulthood.

###### Availability of direct support

Engagement in specific productive activities was dependent on the availability of direct support for individuals within the older two decades of adulthood (i.e., 75–84 and 85 + years). However, this theme was more frequently and extensively discussed by those over the age of 85 years. The availability of direct support was associated with a decline of participation in specific productive activities as explained by FC: “I used to cut my own grass and now I don’t, snow shoveling and all that kind of stuff, I have to have all that done” (85 + years). A participant, FC, explained the importance of the direct support she received from her family as it allowed her to stay in her own home: “been [at home] for over 50 years, if it wasn’t for the kids I’d have had to go into some other place but they come by and anything I need they get, take care of me, so I’m blessed” (85 + years). Taken together, participants within the oldest decades of adulthood experienced a decrease of participation in specific productive activities over the previous 5 years if direct support was available to complete such activities.

## Discussion

This study presents a multidimensional examination of ‘how’ and ‘why’ engagement profiles change throughout older adulthood. Changes in engagement were examined through the frequency of weekly participation (in days per week) across four activity categories, with reasons for these changes being explored using focus groups and semi-structured interviews. Previous work identified that an active engagement in life was a principle component of aging successfully (Carr and Weir, 2017), which was reflected in participants’ maintenance of engagement in personally meaningful and enjoyed activities in the current study.

The direct comparison of changes in participation across activity categories is a novel contribution to the existing literature. Overall, the greatest amount of time was devoted to participation in passive leisure activities, while the least amount of time was spent engaging in productive activities. Additionally, frequency of participation in both social and active leisure activities was similar in the past (5 years ago) and present. The pattern of increased frequency of participation in passive leisure and social activities is supported by previous literature (Gauthier and Smeeding, 2003, 2010; Victorino and Gauthier, 2005; Krantz-Kent and Stewart, 2007), as these are activities preferred and most enjoyed by older adults (Fricke and Unsworth, 2001; Nilsson et al., 2006; Chilvers et al., 2010). Many previous studies combined data across decades of older adulthood, thereby not allowing direct comparisons or the ability to identify points in time where activity patterns changed. The current data extend the previous literature by demonstrating that the frequency of engagement in productive and passive leisure activities differed across the separate decades of older adulthood (‘age’ variable), while there were no age-related differences in participation frequency for social and active leisure activities.

Each activity type was individually examined to provide more granularity of changing engagement patterns. Participants consistently reported decreased participation in productive activities over the previous 5 years, as well as across the three decades of older adulthood, with the 65–74 year-old participants maintaining the highest level of participation. As supported by previous research, participants reported decreases in specific activities such as volunteer work, care for others, home repairs, and heavy housework (Johnson and Schaner, 2004; Fast et al., 2006; Krantz-Kent and Stewart, 2007). Previous literature suggests that domestic activities are maintained at a higher participation rate than other productive activities (Gauthier and Smeeding, 2003; Krantz-Kent, 2005), however, this was only true for light housework.^2^ Caring for others, a specific productive activity, was influenced by one’s responsibilities and enjoyment surrounding the care of grandchildren. If grandchildren required direct care, then patterns of engagement were shifted to incorporate this responsibility, thus *increasing* time spent in productive activities for some individuals. However, participants more commonly identified reasons for *decreased* productive participation, which corresponded to the quantitative findings. For example, the presence of health and physical limitations left participants feeling incapable of continuing such activities, while a sense of freedom from past priorities and a lack of desire to participate supported older adults in choosing preferred engagement activities. In addition, if participants had support to complete productive activities they were content to have others do them, yet still receive the benefit as these activities are typically outcome-based (i.e., cutting the grass; Maier and Klumb, 2005).

The other category that experienced an overall decrease in participation was active leisure, with changes evident across a 5-year time frame (‘time’ variable). This finding supports the limited research available, which suggest leisure time physical activity declines with age (Crombie et al., 2004). Not surprisingly, participants reported walking and gardening between one and four times per week as the activities they participated in most frequently, thereby supporting that most older adults are not meeting recommended guidelines for participation in physical activity (Ashe et al., 2009). Despite the lack of difference among the three decades of older adulthood, participants indicated that unspecified and specified health issues *decreased* active leisure participation. In contrast, other participants stated that remaining and/or becoming physically active was a necessity to overcome or manage these health-related issues, consequently, *increasing* active leisure engagement. This supports the notion that participating to receive health benefits, or to mitigate health limitations, acts as a stimulus for engaging in physical activity (Cohen-Mansfield et al., 2003; Mathews et al., 2010). However, this was only recognized by participants in the youngest decade of older adulthood (i.e., 65–74 year olds). As such, it may be beneficial to use this change in activity participation as a model to promote engagement in active leisure activities for all older adults, as health can evidently be both a barrier and a motivator to participate in physically active leisure.

Participants reported that the frequency of engagement in social activities throughout the decades of older adulthood remained stable. However, preference for specific social activities was impacted by participants’ individual desires, experiencing the death of a friend and/or spouse, and financial constraints. A lack of desire was often grounded in laziness, supporting the notion that (dis)interest often dictates engagement (Crombie et al., 2004; Agahi et al., 2006). Those who had lost friends and family members spoke of a loss of social network, and an increasing desire to stay at home (Bassuk et al., 1999; Andrew, 2005), while widowed participants voiced feelings of social isolation and a reduction in overall activity (Strain et al., 2002). Despite frequency of social participation being maintained, findings suggest an increase in desire and contentment to participate in home-based, passive leisure activities over the previous 5 years of older adulthood (Gauthier and Smeeding, 2003). This supports quantitative findings that identify the social activities with the most frequent participation are those engaged in within a home environment including family/friendship activities, speaking on the phone, and visiting. As such, types of engagement activities may reflect a preference for activities based on the perceived enjoyment of the specific activity (Fricke and Unsworth, 2001; Nilsson et al., 2006; Chilvers et al., 2010). Furthermore, financial constraints may influence participation in specific types of social pursuits, as group activities can be expensive for individuals with modest, fixed incomes.

Similarly, stability of engagement frequency in passive leisure activities was reported. This contradicts the literature that suggests participation in passive leisure pursuits increase with age (Krantz-Kent and Stewart, 2007; Statistics Canada, 2015). However, the present study employed unique methods to examine changes in passive leisure engagement through cross-sectional and retrospective data providing an opportunity for semi-longitudinal trends to be examined. As previous studies have relied solely on cross-sectional data, the current study adds a unique perspective by indicating stable patterns of engagement over a 5-year period within the same individual. Additionally, this study defined passive leisure using Maier and Klumb’s (2005) definition of discretionary activities that are chosen based on one’s preferences and/or abilities. Other studies had broader definitions of leisure that included social, passive leisure, and active leisure activities combined, or that incorporated regenerative and/or personal care activities, such as sleeping or eating (Gauthier and Smeeding, 2003; Krantz-Kent and Stewart, 2007). Therefore, it may be appropriate to speculate that reports of increased participation in passive leisure activities may be confounded by the addition of personal care activities. Taken together, the evidence supports that older adults across three decades of older adulthood remain involved in a wide range of activities on a weekly basis. In fact, 100% of participants engaged in at least one activity from each category (productive, social, active leisure, passive leisure) between one and four times per week. It is, however, important to note that the participant sample included community dwelling older adults who were mostly recruited within public locations. As such, the sample represents individuals who have maintained a connection with the local community, and may not reflect all levels of physical and cognitive functioning, residing within communities that have differing resources, or common engagement practices of various cultures.

Overall, the lay-based conceptual model of successful aging supported engagement in life as a primary element required to age successfully (Carr and Weir, 2017). While participants experienced decreased engagement in productive and active leisure activities, participants likely continued to consider themselves ‘engaged’ as participation in social and passive leisure activities remained stable throughout older adulthood. Furthermore, engagement was maintained despite health-related issues and physical limitations, which supports aging successfully as a continuum where specific criteria are achieved, rather than a dichotomous label (Bowling and Dieppe, 2005; Baker et al., 2009; Young et al., 2009).

The concept of ‘adaptation’ (discussed in Carr and Weir, 2017) coincides with successful aging literature that emphasizes the importance of selection, optimization, and compensation to ensure well-being into older adulthood (Baltes and Baltes, 1990). Burnett-Wolle and Godbey (2007) hypothesized that if health status and physical functioning impacted leisure time activities, optimization and compensation may permit older adults to maintain participation in an adapted fashion depending on how meaningful the activity was to them. Thus, it is speculated that *how* and *why* engagement profiles change throughout older adulthood is reflected within this theory. For example, a decrease in productive and active leisure participation may be a result of the lack of meaning associated with personally completing such activities. Rather, older adults may prefer to optimize the use of internal (i.e., energy) and external (i.e., finances) resources to engage in activities that are found to be more meaningful, such as participating socially. In addition, older adults also encompass the resource of ‘freedom’ and thus can alter engagement profiles according to their abilities and desires, which often included passive leisure pursuits. Ultimately, the overall engagement profile of an older adult may be the result of the individual selecting to participate in activities that are meaningful and appropriate for their abilities and desires, which would result in greater enjoyment during such activities. Therefore, older adults are compensating by altering their engagement patterns with increasing age to ensure the maintenance of active participation, and thus successful aging.

### Practical Implications

Integrated, evidence-based knowledge of ‘how’ and ‘why’ patterns of engagement change throughout older adulthood is a practical tool for both practitioners and policy makers. Establishing rationale for engagement changes in later life, as illustrated in the qualitative inquiry, provides contextual understanding to frequency changes, as depicted through the quantitative data. Such an understanding highlights that changes to frequency of engagement need not be considered solely an adverse adjustment associated with the aging process. For example, while the decrease in productive activity with age is not surprising given the health-related challenges associated with older adulthood, the decrease may be beneficial if viewed as a welcomed change. By minimizing time spent engaging in productive pursuits, older adults have the option to pursue activities that evoke greater enjoyment. Furthermore, reducing productive activities does not minimize the benefit to the older adult, as Maier and Klumb (2005) suggest that the benefit of the productive activity is the outcome (i.e., a prepared meal, a cleaned house), which can be obtained through the task being completed by a third party. Such cessation of productive activity may, in fact, preserve or enhance well-being as older adults choose to allocate personal resources to more meaningful engagement activities suitable for one’s abilities (Burnett-Wolle and Godbey, 2007). By subscribing to these positive notions related to engagement changes in later life, practitioners and policy makers can frame promotional materials, resources, and programing within this understanding of favorable adaptations.

Similarly, older adults experience a decline in the frequency of participation in active leisure pursuits, which was described to be a consequence of health issues and physical limitations, as well as a lack of desire to engage in such activities. These data, however, provide practical applications for mitigating such loss of activity. First, health issues and physical limitations stimulate participation in active leisure activities among adults 65 to 74 years of age. By equipping practitioners with knowledge of active leisure activities suitable and appealing for adults 75 years of age and older, as well as ensuring the availability of appropriate programing, the onset of health issues and physical limitations may be framed as a motivator to increase active leisure pursuits for the associated health benefits among adults 75 years of age and older, as was the case for those in the younger decade. Second, by realizing the stability of the frequency of engagement in social activities throughout older adulthood, there is potential to use the desire and preference for social engagement to increase active leisure activities. For example, by promoting practical changes such as walking with friends rather than visiting within one’s home, or having social programing that includes physical activities, an older adult may be more likely to choose active pursuits that also fulfill one’s social desires.

As with social activities, frequency of passive leisure engagement was stable throughout older adulthood, with both activity types being identified as more desirable with increasing age, and an activity of preference when given freedom of choice and time. However, participants qualitatively identified an unwanted decrease is social engagement coinciding with the death of their spouse or friends. Recognizing this engagement change as unwelcomed, yet inevitable with increasing age, it supports the need to develop social groups and programing for bereaved spouses, or those seeking new social connections in later life. In doing so, social engagement may more likely be maintained, as desired, for those experiencing death of loved ones.

Taken together, engagement profiles of older adults continue to adapt and evolve through the aging process. Despite changes, all participants maintained engagement in at least one activity within each of the four activity categories throughout older adulthood. This represents a well-rounded aging experience that includes rich forms of engagement, and an activity profile that is shaped by the individual.

## Data Availability Statement

The datasets generated for this study are available on request to the corresponding author.

## Ethics Statement

The studies involving human participants were reviewed and approved by University of Windsor Research Ethics Board. The patients/participants provided their written informed consent to participate in this study.

## Author Contributions

All authors listed have made a substantial, direct and intellectual contribution to the work, and approved it for publication.

## Conflict of Interest

The authors declare that the research was conducted in the absence of any commercial or financial relationships that could be construed as a potential conflict of interest.

## Footnotes

1 Productive = Create societal value and are carried out for the purpose of their outcome; **Social** = Provide interactions among others; **Passive Leisure** = Tend not to include a social component and are commonly carried out by a single person without any necessary company; **Active Leisure** = Help with the maintenance of physical functioning and tend to require a larger expenditure of metabolic energy.

2 One of the challenges in interpreting the published literature is that definitions of activities are not consistent. Productive activities have been defined conservatively (e.g., paid/volunteer work, caring for others; Rowe and Kahn, 1997) and liberally (e.g., paid work, housework, active pursuits; Gauthier and Smeeding, 2003).
